# Overexpression of the Mitochondrial Malic Enzyme Genes (*malC and malD*) Improved the Lipid Accumulation in *Mucor circinelloides* WJ11

**DOI:** 10.3389/fmicb.2022.919364

**Published:** 2022-06-22

**Authors:** Abu Bakr Ahmad Fazili, Aabid Manzoor Shah, Mohammed Fahad Albeshr, Tahira Naz, Mohammad Abass Dar, Wu Yang, Victoriano Garre, Khalid Majid Fazili, Eijaz Ahmed Bhat, Yuanda Song

**Affiliations:** ^1^Colin Ratledge Center for Microbial Lipids, School of Agricultural Engineering and Food Science, Shandong University of Technology, Zibo, China; ^2^Department of Zoology, College of Science, King Saud University, Riyadh, Saudi Arabia; ^3^Department of Biotechnology, University of Kashmir, Srinagar, India; ^4^Departamento de Genética y Microbiología, Facultad de Biología, Universidad de Murcia, Murcia, Spain; ^5^Centre de Biologie Structurale (CBS), Univ. Montpellier, CNRS, INSERM, Montpellier, France

**Keywords:** lipid accumulation, *Mucor circinelloides*, oleaginous fungi, malic enzyme, γ-linolenic acid, genetic engineering, health benefits

## Abstract

*Mucor circinelloides* serves as a model organism to investigate the lipid metabolism in oleaginous microorganisms. It is considered as an important producer of γ-linolenic acid (GLA) that has vital medicinal benefits. In this study, we used WJ11, a high lipid-producing strain of *M. circinelloides* (36% w/w lipid, cell dry weight, CDW), to examine the role in lipid accumulation of two mitochondrial malic enzyme (ME) genes *malC* and *malD*. The homologous overexpression of both *malC* and *malD* genes enhanced the total lipid content of WJ11 by 41.16 and 32.34%, respectively. In parallel, the total content of GLA was enhanced by 16.73 and 46.76% in *malC* and *malD* overexpressing strains, respectively, because of the elevation of total lipid content. The fact that GLA content was enhanced more in the strain with lower lipid content increase and vice versa, indicated that engineering of mitochondrial MEs altered the fatty acid profile. Our results reveal that mitochondrial ME plays an important role in lipid metabolism and suggest that future approaches may involve simultaneous overexpression of distinct ME genes to boost lipid accumulation even further.

## Introduction

Microbial lipids have gained a lot of attention because of their ability to replace traditional sources of lipids (Ratledge, 2001; Zhang et al., 2017). The utilization of oils produced by microbes as prospective biofuels and dietary long-chain fatty acid sources has encouraged researchers to thoroughly investigate the accumulation of lipids in oleaginous microorganisms (Ratledge and Wynn, 2002; Ratledge, 2014). When cultivated in the conditions of low nitrogen and high carbon levels, certain filamentous fungi and yeast species minimize the biosynthesis of nucleic acids and proteins, while enhancing the accumulation of lipids by up to 70% (Rodrigues Reis et al., 2019).

*Mucor circinelloides* is regarded as an important oleaginous microorganism because of its utilization as a microbial cell factory to produce several valuable metabolites and natural compounds (Hameed et al., 2020). Moreover, its genome can be easily manipulated with a variety of molecular tools (Nicolás et al., 2018). So far, there have been numerous studies conducted on lipid accumulation mechanisms both at proteomic and genomic levels (Fazili et al., 2022b). Recently, the construction of highly competent *M. circinelloides* strains producing vital fatty acids, such as GLA, dihomo-gamma-linolenic acid, and stearidonic, has been accomplished by the utilization of various genetic and metabolic engineering strategies (Fazili et al., 2022b).

Among different strategies for increasing lipid levels in *M. circinelloides*, genetic engineering of malic enzyme (ME; EC 1.1.1.40) genes stands out as an important candidate. ME is categorized as an oxidative decarboxylase that is involved in the generation of pyruvate from malate *via* oxidative decarboxylation along with the generation of NADPH/NADH (Chang and Tong, 2003; Vongsangnak et al., 2012). Over expression of cytosolic ME gene has been shown to raise the lipid levels by a 2.5-fold in *M. circinelloides* (Zhang et al., 2007). Interestingly, when ME was overexpressed in another study, it did not increase the lipid content. It was noted that, in the former study, plasmids had a self-replicating nature, while later developed a stable strain by incorporation of a desired gene in the precise locus of the genome (Rodríguez-Frómeta et al., 2013). Additionally, the differences between both studies could be explained by the use of genes from different *M. circinelloides* strains. Similar approaches in other fungi have revealed a common trend of lipid increase when ME is overexpressed. Thus, upon genetic engineering of ME in *Rhodotorula glutinis*, a yeast with oil-producing properties, the lipid content increased by 18.74% (Li et al., 2013). A study about manipulation of the cytosolic ME gene in *Mortierella alpina*, where fatty acid accumulation was noted to be enhanced by 30%, further deliberated on the contrasting results about the role of ME in oleaginous microorganisms (Hao et al., 2014b). Moreover, the accumulation of lipids and generation of NADPH has been reported to be repressed when ME inhibitor was used (Wynn et al., 1997).

Recently, our group has investigated the role of cytosolic ME genes, *cmalB*, and *cmalA*, in the accumulation of lipids (Fazili et al., 2022a). Overexpression of *cmalB* and *cmalA* genes increased the ME activity by 6.4- and 9.8-fold, while the lipid content was increased by 5.8 and 23.2%, respectively. All of the studies done so far have focused on cytosolic ME as a mediator of lipid accumulation. While in this study, for the first time, we have probed about the part played by mitochondrial ME in lipid accumulation, revealing that they control both lipid and GLA accumulation. Therefore, the findings of this study highlight an important roadmap aimed at improving lipid accumulation and GLA content.

## Materials and Methods

### Strains and Conditions of the Culture

Genomic DNA of *M. circinelloides* WJ11 strain (CCTCC No. M 2014424) was utilized to amplify *malC* and *malD* genes. A uracil auxotroph of *M. circinelloides* WJ11, named M65, served as recipient strain in the transformation experiments to overexpress mitochondrial ME genes. YPG or MMC media were employed for culture growth, and the temperature was maintained at 26°C (Nicolás et al., 2007; Hussain et al., 2019). When needed, 200 mg/ml of uridine was supplemented to the media. For colonial growth, the pH was adjusted to 3, while for mycelial growth it was set at 4.5. Primary transformants are heterokaryons because of the occurrence of numerous nuclei in the protoplasts. To increase the proportion of transformed nuclei, initial transformants were grown on selective medium over many asexual cycles. Transformation was carried out by the process as defined earlier (Yang et al., 2020).

To generate fungal seed cultures, 500 ml baffled flasks containing 150 ml of Kendrick and Ratledge medium (Kendrick and Ratledge, 1992) were inoculated with approximately10^7^ spores/ml of *Mc-malC-2* (*malC*-overexpression) and *Mc-malD-3* (*malD*-overexpression), and *Mc-mal-2076* (control) strains. Cultures were grown for 24 h at 28°C and 150 rpm. The whole seed culture was poured into a 2 L fermenter (BioFlo/CelliGen 115, New Brunswick Scientific, Edison, NJ, United States) containing 1.5 L of modified Kendrick and Ratledge medium with 80 g glucose per liter. The fermenter was set at following parameters: temperature = 28°C, aeration = 0.5 volume of air per volume of fermenter per minute (v/v min^–1^), and shaking = 700 rpm. Furthermore, 2M NaOH and 2 M HCL were utilized for automatic maintenance of pH. For all cloning experiments, *Escherichia coli* DH5α was employed and recombinant plasmids were constructed using basic plasmid pMAT2075 (Yang et al., 2020). For propagation and selection of colonies, lysogenic broth, and agar plates (100 mg/L ampicillin) were utilized (Hanahan, 1983).

### Plasmid Construction

For the construction of *malC* and *malD* overexpressing plasmids, a plasmid named pMAT 2075 was used as a vector. The plasmid pMAT2075 contained *pyrF* gene, used as a selectable marker, and the strong *zrt1* promoter (P*zrt1*) both flanked by downstream and upstream *carRP* sequences that allowed homologous recombination, leading to chromosomal integration (Zhao et al., 2016). Isolation of *malC* and *malD* genes from the WJ11 genome was done by PCR amplification using the primer pair’s *malC*-1F-*Xho*I/*malC*-1R-*Xho*I and *malD*-1F-*Xho*I/*malD*-1R-*Xho*I, respectively (Supplementary Table 1). The resulting PCR fragments were digested with *Xho*I and cloned in plasmid pMAT2075 previously digested with *Xho*I (One-step cloning kit, Takara), giving plasmids pMAT2075-malC and pMAT2075-malD that harbored *malC* and *malD*, respectively, under P*zrt1*. Recombinant plasmids were propagated using competent *E. coli* cells. To confirm the correct constructions of the plasmid, the plasmid inserts were PCR amplified and sequenced. The whole overexpressing constructs were released from the recombinant plasmids by digestion with *Sma*I and used to transform M65 protoplasts. Albino transformants were selected, because integration in *carRP* locus generated albino colonies whereas non-integration produced yellow colonies (Rodríguez-Frómeta et al., 2013).

### Preparation of Genomic DNA

For the extraction of DNA, *M. circinelloides* was grown in Kendrick and Ratledge medium at the following conditions: time = 3 days, temperature = 28°C, and shaking = 150 rpm. Mycelium was harvested using a Buchner funnel under reduced pressure and was washed three times with distilled water. Extraction of genomic DNA was performed using the DNA Quick Plant System kit (Tiangen Biotech Co., Ltd.).

### Reverse Transcription-Quantitative PCR (RT-qPCR) to Analyze the Gene Expression

Mycelium was ground under liquid nitrogen and trizol was employed for total RNA extraction. Isolated RNA was transformed into cDNA by using the reagent kit of PrimeScript™ RT (Takara) in accordance with the instructions of the manufacturing company. Reverse transcription-quantitative PCR (RT-qPCR) was performed with the help of primer pair’s *malC*-2-F/*malC*-2-R and *malD*-3-F/*malD*-3-R for *Mc-malC-2* and *Mc-malD-3* strains, respectively (Supplementary Table 1). The LightCycler 96 Instrument (Roche Diagnostics GmbH, Switzerland) and FastStart Universal SYBR Green Master mix (ROX) were utilized for RT-qPCR. *Actin* gene was used as a housekeeping gene and quantitative analysis of data was performed by 2^–ΔΔ*Ct*^ procedure (Naz et al., 2021).

### Measurement of Nitrogen and Glucose Concentration in Culture Medium

The ammonium concentration in the culture medium was determined using the indophenol method (Chaney and Marbach, 1962). In accordance with the manufacturer’s guidelines, glucose concentration was measured using a glucose oxidase Perid-test kit (Shanghai Rongsheng Biotech Co., Ltd.).

### Analysis of Cell Dry Weight and Lipid Accumulation

**FIGURE 1:**
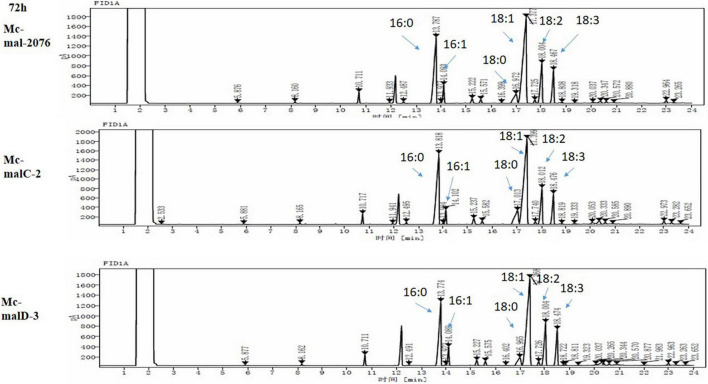
Chromatogram of *Mc-mal- 2076* (control), *Mc-malC-2*, and *Mc-malD-3* strains at 72 h.

Harvesting of biomass samples from fungus was done by a suction-filtration technique using a Buchner funnel. To get rid of supplementary medium from harvested mycelia, it was washed with distilled water three times. This mycelia was then kept at −80°C overnight, followed by freeze-drying. Then, cell dry weight (CDW) was calculated gravimetrically (Hussain et al., 2019) and, a minorly modified Folch method (Folch et al., 1957) was employed to perform lipid extraction, which was followed by fatty acid methyl esters (FAMEs) analysis using gas chromatography (GC). Each chromatographic peak was determined by comparing it to a fatty acid standard curve, and for respective peaks, corresponding fatty acids were determined (Figure 1).

### Determination of Malic Enzyme Activity

The Zhang et al. (2007) method was employed to prepare cell extracts. The ME activity assay was performed as described by Hsu and Lardy (1967) with minor modifications. During the process, 100 mg of fresh mycelium was washed, frozen, and crushed. To it, 500 μl of extraction buffer (50 mM Tris-HCL, pH 7.5 with 20% glycerol w/w) was added. This was followed by the addition of 5 μl DTT (100 mM) and 5 μl Benzamidine (100 mM). This solution was then shaked by vortexing and kept on ice for 30 min and stirred at intervals in the vortex. The supernatant was then centrifuged at 13,000 rpm for 30 min at 4°C. Then, the supernatant was transferred to a clean eppendorf tube and used for an enzyme activity assay. An activity assay was performed using a reaction mixture containing 500 μl of 0.4 M Triethanolamine Buffer, 50 μl of 30 mM L-malate (stored at −20°C), 100 μl of 0.12 M MnCl_2_, 200 μl of 3.4 M TPN (stored at −20°C), and 50 μl of distilled water. This reaction mixture was added to the cuvette, along with 100 μl of the enzyme extract and the cuvette was quickly placed in the spectrophotometer and the measurement was started at 340 nm. Enzymatic activity was measured for about 5 min, and the data were recorded every 30 s. The Bradford method (Bradford, 1976) was used to determine the protein concentrations, using BSA as a standard.

### Statistical Analyses

The experiments were performed using three independent replicates. One-way/two-way ANOVA (wherever applicable) followed by multiple comparison tests *via* GraphPad Prism (version 7, San Diego, CA) was performed for statistical analysis. The value of *p* < 0.05 was considered to be significantly different.

## Results

### Overexpression of *malC* and *malD* in *Mucor circinelloides*

**FIGURE 2:**
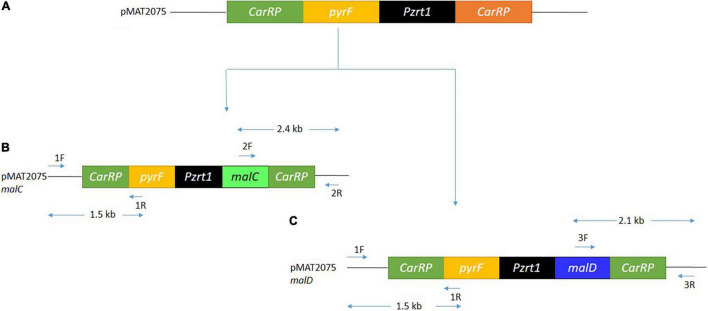
The overexpression of *malC* and *malD* gene. **(A)** Structure of the relevant region of pMAT2075 plasmid. **(B)** Structure of pMAT2075-*malC* plasmid, showing primer positions, and expected size fragments after PCR. **(C)** Structure of pMAT2075-*malD* plasmid, showing primer positions, and expected size fragments after PCR.

The desired genes *malC* (scaffold00188.29) and *malD* (scaffold00014.40) were obtained from the genomic records of *M. circinelloides* WJ11 (Tang et al., 2015b). A plasmid named pMAT2075 (carrying the strong promoter *zrt1* and *pyrF* gene as a selectable marker) was utilized to overexpress these genes (Figure 2A; Yang et al., 2020). The recombinant plasmids for *malC* and *malD* genes were named pMAT2075*-malC* and pMAT2075*-malD*, respectively. Plasmids pMAT2075*-malC* and pMAT2075*-malD* were transformed into *M. circinelloides* WJ11 uracil auxotrophic strain M65. In the recombinant plasmids, the pyrF gene was utilized to complement uridine auxotrophy in the M65 strain. *Mc-malC-2* and *Mc-malD-3* were selected as independent transformants for *malC* and *malD* genes, respectively. An *Mc-mal-2076* strain having wild-type characteristics was used as a control strain.

**FIGURE 3:**
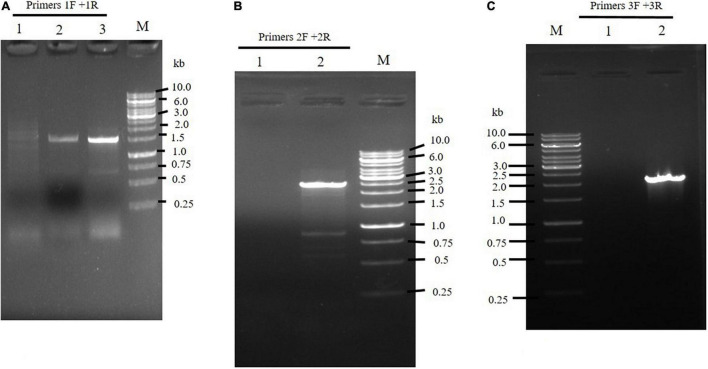
Confirmation of the integration of overexpressing constructs by **(A)** the use of 1F and 1R primers to amplify pyrF and 5′ carRP region showing a band size of 1.5 kb in *Mc-malC-2*, and Mc-cmalD-3 strains. Lane M: Marker. Lane 1 designates the control. Lanes 2 and 3 illustrates pyrF gene presence in *Mc-malC-2* and Mc-cmalD-3 strains, respectively. **(B)** The use of 2F and 2R primers to amplify 3′ carRP region and *malC* gene, showing a band size of 2.4 kb in *Mc-malC-2* strain. Lane M: Marker, Lane 1 designates the control, Lane 2 shows PCR amplification of 3′ carRP region and *malC* gene. **(C)** The use of 3F and 3R primers to amplify 3′ carRP region and *malD* gene, showing a band size of 2.1 kb in *Mc-malD-3* strain. Lane M: Marker, Lane 1 designates the control, Lane 2 shows PCR amplification of 3′ carRP region and *malD* gene.

The integration of DNA fragments onto the genome of *M. circinelloides* was verified through PCR. Primer pairs 1F and 1R generating 1.5 kb band size (Figures 2B,C, 3A) confirmed *pyrF* gene integration into genome of *Mc-malC-2* and *Mc-malD-3* strains, respectively. The control strain did not show any PCR bands as shown in Figure 3A. Integration of the *malC* gene in the *carRP* locus of M65 was authenticated by primer pairs 2F and 2R, yeilding an expected band size of 2.4 kb (Figures 2B, 3B). Similarly, the integration of the *malD* gene in the *carRP* locus of M65 was confirmed with the help of primer pairs 3F and 3R, getting an expected band size of 2.1 kb (Figures 2C, 3C). For the control strain, no bands were observed as shown in Figures 3B,C.

### The Expression Levels of *malC* and *malD* Genes in Overexpressing Strains

**FIGURE 4:**
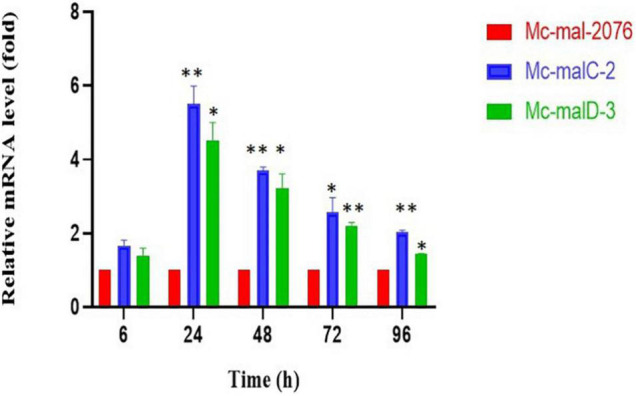
Relative mRNA levels determined by reverse transcription-quantitative PCR (RT-qPCR) of *malC* and *malD* genes in *Mc-malC-2* and *Mc-malD*-3, respectively, normalized against their corresponding levels in the control strain *Mc-mal-2076*. Three biological replicates were used to calculate the respective values. Standard error of the mean (SEM) is indicated by error bars. Significant differences are indicated by asterisks: *p < 0.05 and **p < 0.01.

An RT-qPCR was performed to estimate the mRNA levels of *malC* and *malD* genes in respective *Mc-malC-2* and *Mc-malD-3* strains and control strain *Mc-mal-2076*. It was carried out at 6, 24, 48, 72, and 96 h of growth using primer pairs *malC*-3-F/*malC*-3-R and *malD*-3-F/*malD*-3-R for *malC* and *malD* genes, respectively (Supplementary Table 1). Messenger RNA (mRNA) levels were noted to be increased by 5.5- and 4.5-fold for *malC* and *malD* genes, respectively. Though the level of expression was observed to be increased rapidly from 6 to 24 h, a constant decline was noted in the mRNA levels after 24 h. However, the overexpression of *malC* and *malD* genes in the recombinant strains was validated by the fact that across all hours their mRNA levels were sustained at higher levels in comparison with the control strain (Figure 4). As a reference, Mc-mal-2076 was used to analyze expression levels at specified time points and its relative expression value was taken as 1.

### Malic Enzyme Activity in *malC* and *malD* Overexpressing Strains

**FIGURE 5:**
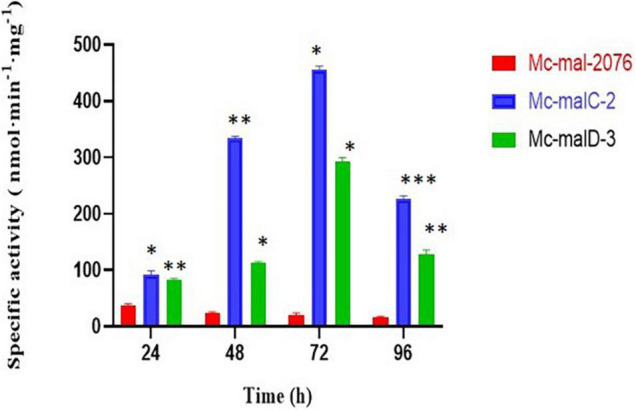
Malic enzyme (ME) specific activity in *Mc-mal-2076*, *Mc-malC-2*, and *Mc-cmalD-3* strains. Three biological replicates were used to calculate the respective values. SEM is indicated by error bars. Significant differences are indicated by asterisks: *p < 0.05, **p < 0.01, and ***p < 0.001.

The accumulation of *malC* and *malD* mRNAs observed in the overexpressing strains was linked to an elevation of ME specific activity in comparison to the control strain. As compared with the control strain, which showed a decrease in ME activity throughout the culture, both recombinant strains showed a constant increase of ME activity that picked at 72 h (Figure 5). Thus, a 22.9- and 14.7-fold increase of the ME specific activity was noted in *Mc-malC-2* and *Mc-malD-3*, respectively, in comparison with the control strain.

### The Effects of *malC* and *malD* Overexpression on Cell Growth and Lipid Accumulation

**FIGURE 6:**
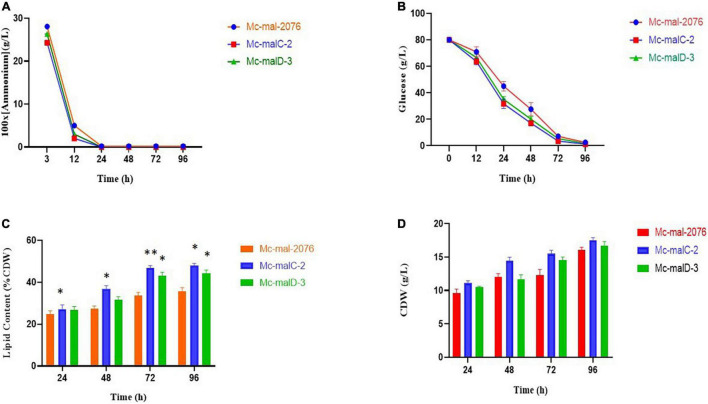
Accumulation of lipids and cell growth in overexpressing transformants (*Mc-malC-2* and *Mc-cmalD-3*) and control strain (*Mc-mal-2076*). **(A)** Ammonium consumption profile, **(B)** glucose consumption profile, **(C)** lipid accumulation profile, and **(D)** cell dry weight (CDW). Three biological replicates were used to calculate the respective values. SEM is indicated by error bars. Significant differences are indicated by asterisks: *p < 0.05 and **p < 0.01.

Analysis of growth and lipid accumulation in control and recombinant strains was carried out to determine the effect of ME overexpression. Consumption rates of ammonium and glucose were observed to be similar among the control and overexpressing strains throughout the culture, but their utilization was observed to be slightly slower in the control strain (Figures 6A,B). After the depletion of nitrogen at about 12–24 h, rapid lipid accumulation in the three strains started (Figures 6C,D). In *Mc-malC-2* and *Mc-malD-3* strains, compared with the control strain *Mc-mal-2076*, lipid accumulation at 72 h was increased by 41.16% (from 34% in control strain to 48% in overexpressing strain) and 32.34% (from 34% in control strain to 45% in overexpressing strain), respectively (Figure 6C). For the overexpressing and control strains, there was a constant increase in CDW over the progressing hours and it was noted to be maximum at 96 h (Figure 6B). Due to the overall increase in lipid content in recombinant strains, the GLA in biomass was noted to be increased by 16.73% (from 2.63 to 3.07%) and 46.76% (from 2.63 to 3.86%) in *Mc-malC-2* and *Mc-malD-3* strains, respectively (Table 1). Table 1 shows the total fatty acid profile of *Mc-malC-2*, *Mc-malD-3*, and *Mc-mal-2076* strains and GC chromatograms of the respective strains at 72 h are shown in Figure 1.

**TABLE 1:** Fatty acid composition at 24, 48, 72, and 96 h (relative%, w/w) in *Mc-mal- 2076*, *Mc-malC-2*, and *Mc-malD-3* strains.

Strains	Time (h)	16:0	16:1	18:0	18:1	18:2 (LA)	18:3 (GLA)
*Mc-mal-2076*	24	17.44 ± 1.20	2.61 ± 0.35	4.90 ± 0.21	39.10 ± 1.74	11.01 ± 0.71	11.59 ± 0.20
	48	21.18 ± 1.71	2.92 ± 0.21	5.18 ± 1.29	41.12 ± 2.23	10.74 ± 0.44	9.00 ± 1.10
	72	22.55 ± 1.52	3.41 ± 0.11	4.62 ± 0.44	41.88 ± 2.91	11.09 ± 0.56	7.76 ± 0.32
	96	22.59 ± 1.81	4.00 ± 1.11	3.33 ± 0.31	42.94 ± 1.67	12.41 ± 0.30	7.71 ± 0.75
*Mc-malC-2*	24	19.59 ± 1.63	1.59 ± 0.5	6.80 ± 0.42	34.33 ± 2.34	14.96 ± 1.22	14.04 ± 1.21
	48	23.71 ± 1.23	2.01 ± 0.65	8.05 ± 1.33	37.87 ± 2.80	9.73 ± 0.32	7.80 ± 0.21
	72	24.89 ± 2.11	2.42 ± 0.34	6.97 ± 0.87	40.43 ± 1.91	9.10 ± 0.76	6.40 ± 0.45
	96	24.56 ± 2.73	2.96 ± 0.21	5.89 ± 0.40	41.14 ± 1.56	9.79 ± 0.63	6.46 ± 0.63
*Mc-malD-3*	24	16.96 ± 1.22	2.66 ± 0.21	4.65 ± 0.34	39.62 ± 0.92	12.18 ± 0.54	14.32 ± 0.38
	48	20.52 ± 2.10	2.61 ± 0.13	5.46 ± 0.75	40.39 ± 1.78	11.53 ± 0.45	10.55 ± 0.76
	72	20.16 ± 2.07	3.43 ± 0.87	3.91 ± 0.92	41.82 ± 1.95	11.62 ± 0.72	8.59 ± 0.87
	96	21.68 ± 1.99	3.34 ± 0.56	4.28 ± 0.44	40.13 ± 2.22	10.42 ± 0.88	7.23 ± 0.15

*The values are means ± standard deviations (SDs) of three independent experiments.*

## Discussion

The high lipid generating filamentary fungus *M. circinelloides* WJ11 serves as a microorganism model for the study of lipid accumulation because of its easy cultivation, production of high amount of lipids, and availability of genetic tools (Mohamed et al., 2021). There have been several studies about the metabolism, proteomics, and genomics of *M. circinelloides*, which makes it convenient to enhance and influence its lipid content *via* genetic and metabolic engineering approaches (Tang et al., 2015b,^2016^). The biosynthesis of lipids takes place in cytosol, needing acetyl-CoA and enough NADPH as an important reducing agent in sufficient quantity. The generation of acetyl-CoA occurs from citrate while ME acts as one of the important source of NADPH (Ratledge, 2014).

**FIGURE 7:**
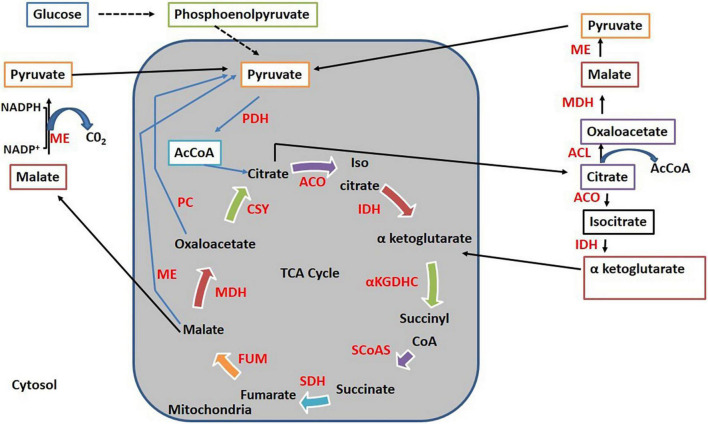
Cycling of malate *via* different pathways. ACL, ATP, citrate lyase; ME, Malic enzyme; PC, pyruvate carboxylase; PDH, pyruvate dehydrogenase; AcCOA, Acetyl CoA; MDH, malate dehydrogenase; CSY, citrate synthase; ACO, aconitase; IDH, isocitrate dehydrogenase; αKGDHC, α-ketoglutarate dehydrogenase complex; SCoAS, succinyl-CoA synthetase; SDH, succinate dehydrogenase; FUM, fumarase.

It has been reported that when mitochondrial ME genes were overexpressed in *Mortierella alpina*, it resulted in enhanced arachidonic acid production (Hao et al., 2014a). Mitochondrial ME found in diatom microalgae *P. tricornutum* has been shown to play a positive role in the biosynthesis of lipids (Xue et al., 2015, 2016; Zhu et al., 2018). The role of mitochondrial ME in the biosynthesis of lipids can be partially described by lipid regulation by endoplasmic reticulum and mitochondria organelle connections (Rowland and Voeltz, 2012). Figure 7 shows how cytosolic ME cycles malate from various direct and indirect pathways. First, it cycles malate synthesis implicitly from exported citrate that undergoes conversion into oxaloacetate and malate *via* ATP: citrate lyase and malate dehydrogenase. Second, it cycles malate produced by mitochondrial malate export. Moreover, by generating pyruvate from malate inside the mitochondria, ME can also initiate the pyruvate cycle completely within the mitochondria. The generation of α-ketoglutarate from exported citrate *via* isocitrate presents an alternative cycle toward the conversion of citrate into α-ketoglutarate (Pongratz et al., 2009). It can be concluded that all of these pathways are interconnected and they can be reasoned as a mechanism for the transmission of mitochondrial reducing equivalents to the cytosol in the form of NADPH, which is used for lipid biosynthesis (Pongratz et al., 2009). This clearly explains that in the present study, overexpression of mitochondrial ME genes, *malC* and *malD*, resulted in the formation of more malate in cytosol, which consequently lead to the formation of more NADPH from cytosolic ME, resulting in more lipid accumulation. Interestingly, a study about the role of mitochondrial ME in *Yarrowia lipolytica* has revealed that it has no role in lipid accumulation (Dulermo et al., 2015). However, in the case of *Yarrowia lipolytica*, cytosolic ME does not exist, whereas in the present study, involvement of mitochondrial ME in the accumulation of lipids appears to be *via* cytosolic ME.

During our study, it was established that across different strains, glucose and nitrogen consumption patterns were almost similar (Figures 5A,B). Generally, in oleaginous microorganisms, the accumulation of lipids is initiated in nitrogen-limited conditions (Fazili et al., 2022a). The same pattern was observed for *malC* and *malD* overexpressing strains with no substantial variation in lipid levels preceding the depletion of nitrogen as compared with the control strain. Lipid accumulation was enhanced more significantly in recombinant strains in comparison with the control strain after nitrogen depletion at about 24 h. Thus, the overexpression of *malC* and *malD* produced an increase of 41.16 and 32.34% in lipid production at 72 h of growth, respectively. These results suggest that the negative regulation by nitrogen on lipid biosynthesis is intact in the overexpressing mutants, and the elevation of lipid levels might be due to the higher levels of NADPH. Even though lipid content was enhanced more in *Mc-malC-2* strain compared to *Mc-malD-3* strain, interestingly, the GLA content was noted to be increased more in *Mc-malD-3* (46.76%) in comparison with *Mc-malC-2* (16.73%). From this, it can be inferred that engineering of mitochondrial ME genes does not simply influence the lipid levels but also alters the fatty acid profile. Zhang et al. (2007) have suggested that the elevation of ME activity is linked with NADPH generation that strongly influences fatty acid desaturase activity, thereby increasing the polyunsaturated fatty acid content, including GLA. A study about the role of ME in *P. tricornutum* has revealed that overexpression of ME significantly increases the activity of fatty acid desaturase, thereby enhancing the polyunsaturated fatty acid accumulation (Zhu et al., 2018). Overexpression of ME in *E. coli* has also been shown to influence the fatty acid composition where C16:1 was noted to be increased by 5.6-fold (Lv et al., 2016). Taken together with previous studies, the findings of current study indicate that the desaturase activity of ME is gene dependent. It can be suggested that, even though overexpression of the *malD* gene enhances lipid accumulation less compared with *malC*, but *malD* influences fatty acid desaturation more strongly compared with *malC*.

The ME activity in *malC* and *malD* overexpressing strains was significantly increased up by 22.9- and 14.75-fold, respectively, while lipid accumulation increased only by 41.16 and 32.34%, respectively. Based on these results, it can be interpreted that, other than ME, there are various other factors that impact the accumulation of lipids in *M. circinelloides*. This is in accordance with the previous studies that indicate that ME is not the only rate-limiting enzyme; nonetheless, it is important for fatty-acid synthesis (Rodríguez-Frómeta et al., 2013; Hao et al., 2014b). The ME activity in recombinant strains might also be impacted by metabolic route of carbon, and it is asserted that acetyl-CoA has a more significant role than presumed before (Zhang et al., 2007). During the synthesis of fatty acids in *M. circinelloides*, endogenic leucine metabolism generating acetyl-CoA has been supposed to be one of the rate-limiting phases (Rodríguez-Frómeta et al., 2013). This emphasized that acetyl-CoA might acts as an intersection between the synthesis of fatty acid residues and the metabolism of amino acids. Similarly, acetyl-CoA and NADPH for lipid metabolism in *M. alpina* have been postulated to be furnished during the metabolism of tyrosine and phenylalanine (Wang et al., 2013). Genetic engineering of *Yarrowia lipolytica* to industrially produce eicosapentaenoic acid, increased its content of fatty acids up to 56.5% (Xue et al., 2013). This indicated that fatty acid content, and not only fatty acid composition, is determined by the flux of carbon during fatty acid biosynthesis.

An investigation about *Aspergillus oryzae* has reported that under nitrogen limitation conditions, the biosynthesis of malic acids is significantly enhanced (Knuf et al., 2013). *A. oryzae* has emerged as an important source of malic acid production in the industrial sector because of increase in hydrolytic secretory enzymes maintained at low pH (Machida et al., 2005). In the case of *M. alpina*, it has been reported that, under nitrogen limitation, when malic acid accumulates intracellularly, its responded by an elevation in ME activity that was repressed by low pH. While low pH corresponds to high ME activity, it is not conducive to fatty acid accumulation in *M. alpina*. Intriguingly, in some cases, various isoforms of ME in *M. circinelloides* were reported to be unrelated to the accumulation of lipids (Song et al., 2001). Some studies have reported that ME may not be the most important enzyme for the production of NADPH, but nevertheless it does have a contribution to the production of fatty acids (Tang et al., 2015a; Zhao et al., 2015). Results of our study further emphasize this observation, that despite the fact that ME is important in lipid accumulation, it is not the lone enzyme that limits accumulation of lipids. Moreover, ME in *M. circinelloides* is encoded by five different genes and each gene has a varying role related to the accumulation of lipids. In our previous study, it has been noted that *cmalA*, a cytosolic ME gene increased the accumulation of by 23.2%, while other cytosolic ME gene influenced the accumulation of lipids only by 5.8% (Fazili et al., 2022a). Combined with results reported in current study, it can be concluded that the role of ME in lipid accumulation is gene dependent. Further scope, about the contribution of ME in lipid accumulation can involve co-overexpression of different pairs of ME genes.

## Conclusion

This study strongly recommends that mitochondrial ME is a key player in the biosynthesis of lipids in *M. circinelloides* and improves our comprehension of the molecular mechanisms that drive the accumulation of lipids in oleaginous fungi. It can be concluded that *malC* gene induces lipid accumulation to a greater extent in comparison with the *malD* gene. Interestingly, the *malD* gene influences GLA synthesis more significantly compared with *malC* gene. Although, our group has previously investigated about the part played by cytosolic ME genes in lipid accumulation, this is the first study reporting about the enhancement of lipid accumulation in *M. circinelloides* WJ11 because of overexpression of mitochondrial ME genes. Moreover, the co-expression of both mitochondrial genes *malC* and *malD* may yield a promising industrial fungal strain that could create a technological breakthrough in GLA rich lipid production.

## Data Availability Statement

The original contributions presented in the study are included in this article/Supplementary Material, further inquiries can be directed to the corresponding author.

## Author Contributions

AF performed all the experiments and wrote the initial draft of the manuscript. AS, MA, TN, MD, and WY were involved in experimental designing and data analysis. VG, KF, and EB improved the draft of the manuscript. YS designed, supervised, and coordinated the project. All authors contributed to the article and approved the submitted version.

## Conflict of Interest

The authors declare that the research was conducted in the absence of any commercial or financial relationships that could be construed as a potential conflict of interest.

## Publisher’s Note

All claims expressed in this article are solely those of the authors and do not necessarily represent those of their affiliated organizations, or those of the publisher, the editors and the reviewers. Any product that may be evaluated in this article, or claim that may be made by its manufacturer, is not guaranteed or endorsed by the publisher.
