# Supplementary material for: Overexpression of the Mitochondrial Malic Enzyme Genes (malC and malD) Improved the Lipid Accumulation in Mucor circinelloides WJ11
Source: Front Microbiol. 2022 Jun 22;13:919364. doi: 10.3389/fmicb.2022.919364 (PMC9260706; doi:10.3389/fmicb.2022.919364)
Supplement: Supplementary file 1 [file Table_1.docx]

**Table S1.** Sequences of primers utilized in paper titled “Overexpression of the Mitochondrial Malic Enzyme Genes (*malC* and *malD*) Improved the Lipid Accumulation in *Mucor circinelloides* WJ11”

| Primer name | Primer sequence (5’-3’) |
| --- | --- |
| *malC*-1F-*Xho*I | CAAAATAACTAAATTACGTAGCTAGCCTCGAGATGTTAACTACAAGATCATTATCG |
| *malC*-1R-*Xho*I | CTCATCTTTCCCTGTCTGCCTCGAGCTAATGGGAAGGACTATGCACC |
| *malD*-1F-*Xho*I | CAAAATAACTAAATTACGTAGCTAGCCTCGAGATGAATCTTTATAAATCCACCCATCTC |
| *malD*-1R-*Xho*I | CTCATCTTTCCCTGTCTGCCTCGAGTTACTCATAGCTAGATGTCCACATGCG |
| 1F | GATAAGCATAAACCAGATCTGC |
| 1R | GAGATCTCGACGTATTCAGCG |
| 2F | GGTAAACTCAGTTTGGTAATGTATC |
| 2R | GTATCTGACATAGTCGAGCTTG |
| 3F | GGTGCAGTCATTTTATCGG |
| 3R | GTATCTGACATAGTCGAGCTTG |
| *malC*qPCR-F | TCCCTGGCCTTGGTCTTGGT |
| *malC*qPCR-R | GGGGACGTACTCTGGGACGA |
| *malD*qPCR-F | AGGCGCCGTCATCTTGTCAG |
| *malD*qPCR-R | CGATCGCCACGATCAGCAGT |
| Actin-F | GATGAAGCCCAATCCAAGAGAGGT |
| Actin-R | TCTTCTCCGGTTGGACTTGGG |

* Restriction enzyme sites (6 bp nucleotides) are underlined.
